# Supplementation of Lipoic Acid, Zinc and Clopidogrel Reduces Mortality Rate and Incidence of Ventricular Arrhythmia in Experimental Myocardial Infarction

**DOI:** 10.3389/fphys.2021.582223

**Published:** 2021-03-17

**Authors:** Enas Abdel-Hady, Fatma Mohamed, Mona Ahmed, Mohamed Abdel-Salam, Mahmoud Ayobe

**Affiliations:** Department of Physiology, Faculty of Medicine, Ain Shams University, Cairo, Egypt

**Keywords:** lipoic acid (LA), zinc, clopidogrel, myocardial infarction, isoproterenol (ISO)

## Abstract

Despite the significant advances in management of coronary heart diseases, myocardial infarction (MI) is still associated with a high mortality rate. The present study was planned to investigate the possible protective effects of the anti-oxidants lipoic acid and zinc sulfate as well as the anti-platelet clopidogrel on cardiac dysfunction in experimental isoproterenol (ISO)-induced MI, aiming at achieving useful means for protection and therapy against MI. Wistar rats of both sexes were allocated into five groups: control, untreated MI and MI pre-treated with lipoic acid, zinc, or clopidogrel. All rats were subjected to ECG recording and measurement of plasma levels of troponin I, creatine kinase-MB (CK-MB) unit, triglycerides and total cholesterol. The hearts were isolated and studied on Langendorff preparation for assessment of intrinsic cardiac activities. The results revealed that the percent mortality was markedly reduced upon pre-treatment and the total arrhythmia was also decreased except for the zinc pre-treated rats. The ST-segment elevation was significantly reduced and the plasma levels of CK-MB were only decreased in lipoic acid and clopidogrel pre-treated rats with variable hypolipidemic effect. Hearts of clopidogrel pre-treated rats showed augmented inotropic activity both basal and in response to β-adrenergic stimulation. While zinc pre-treated hearts revealed improved rate of contraction and increased myocardial flow rate. Overall, these results indicate that lipoic acid, zinc and clopidogrel were variably effective in modifying the ISO-induced MI insults and offered partial protection against experimental myocardial damage.

## Introduction

Acute myocardial infarction (AMI) is one of the acute coronary syndromes (ACS) which are conditions associated with myocardial ischemia, and despite significant advances in the management of coronary heart diseases, myocardial infarction is still associated with a high mortality rate (Goldberger et al., 2015). In a previous study, we established that rats subjected to isoproterenol (ISO)-induced myocardial infarction (MI) developed high incidence of mortality and arrhythmia, together with bradycardia, systolic dysfunction and hyperlipidemia (Mohamed et al., 2012).

Oxidative stress and reactive oxygen species (ROS) have been implicated in the pathophysiology of MI (Rajadurai and Prince, 2006). Also, changes in trace element levels have been detected in ACS patients, higher levels of Fe and Cu and lower levels of Se and Zn correlated well with the degree of myocardial damage (Altekin et al., 2005). Further, functional alterations in mitochondria of cardiac muscle cells, with reduced oxygen consumption and ATP synthesis as well as energy imbalance, have been reported in MI, the alterations reaching a maximum at the onset of infarction (De Sánchez et al., 1997). In a previous study, we demonstrated the role of insulin, the high-energy substrate ATP and the nitric oxide (NO) precursor L-arginine in partial modifying the ISO-induced MI insults as well as offering partial protection against ISO-induced myocardial damage (Mohamed et al., 2013).

Alpha lipoic acid (α-LA) is water and fat soluble sulfur-containing compound that possesses potent anti-oxidant properties (Wada et al., 1997). α-LA was reported to be required for oxidative decarboxylation of pyruvate to acetyl CoA, the critical step bridging the gap between glycolysis and citric acid cycle (Reed, 1998). α-LA supplementation was reported to have favorable effects on cellular redox, and has been shown to decrease lipid peroxidation and cellular production of ROS (Suh et al., 2004). In fact, α-LA and its reduced form (dihydrolipoic acid) have been referred to as universal anti-oxidants that function in both aqueous and membranous phases (Handelman et al., 1994), and are considered to be one of the most powerful biological anti-oxidant systems (Moini et al., 2002).

Zinc is a component of several metalloenzymes with redox capacity, which gives them anti-oxidizing and anti-reactive oxygen radical action (e.g., superoxide dismutase) (Cikim et al., 2003). Further, zinc is known to induce the production of metallothionein, which is an excellent scavenger of ^⋅^OH (Powel, 2000). Metallothionein improves mechanical properties of the heart and diminishes the incidence of fatal ventricular arrhythmia (Karagulova et al., 2007). Beattie and Kwun (2004) stated that zinc and metallothionein deficiency might be a risk factor for ischemic heart disease as their deficiency in endothelium facilitates certain oxidative reactions.

Furthermore, ACS were reported to occur as a result of atherosclerotic and thrombotic processes in the coronary circulation (Corti et al., 2002), and there is well-established role of platelets in atherosclerosis and atherothrombosis (Bhatt and Topol, 2003), pointing to the possible contribution of platelets to myocardial ischemia. Clopidogrel is a strong platelet anti-aggregating agent which was discovered in 1986, and was documented to be a potent dose-dependent anti-aggregating and anti-thrombotic compound in several animal species and in humans (Herbert et al., 1993). Moreover, clopidogrel was reported to improve endothelial NO bioavailability, and, as well, reduces markers of platelet activation, pointing to it as being a promising vasoprotective agent (Heitzer et al., 2006).

The present study was planned to investigate the possible protective effects of the anti-oxidants lipoic acid and zinc sulfate as well as the anti-platelet clopidogrel on cardiac dysfunction in rat hearts with ISO-induced MI aiming at achieving useful means for protection and therapy against MI.

## Materials and Methods

### Experimental Protocol

All procedures involving experimental animals were performed in strict accordance with the recommendations in the Guide for the Care and Use of Laboratory Animals of the National Institutes of Health (NIH, 8th edition, revised 2011). The experimental protocol was also approved by the Research Ethics Committee (REC), Faculty of Medicine, Ain Shams University.

**FIGURE 1:**
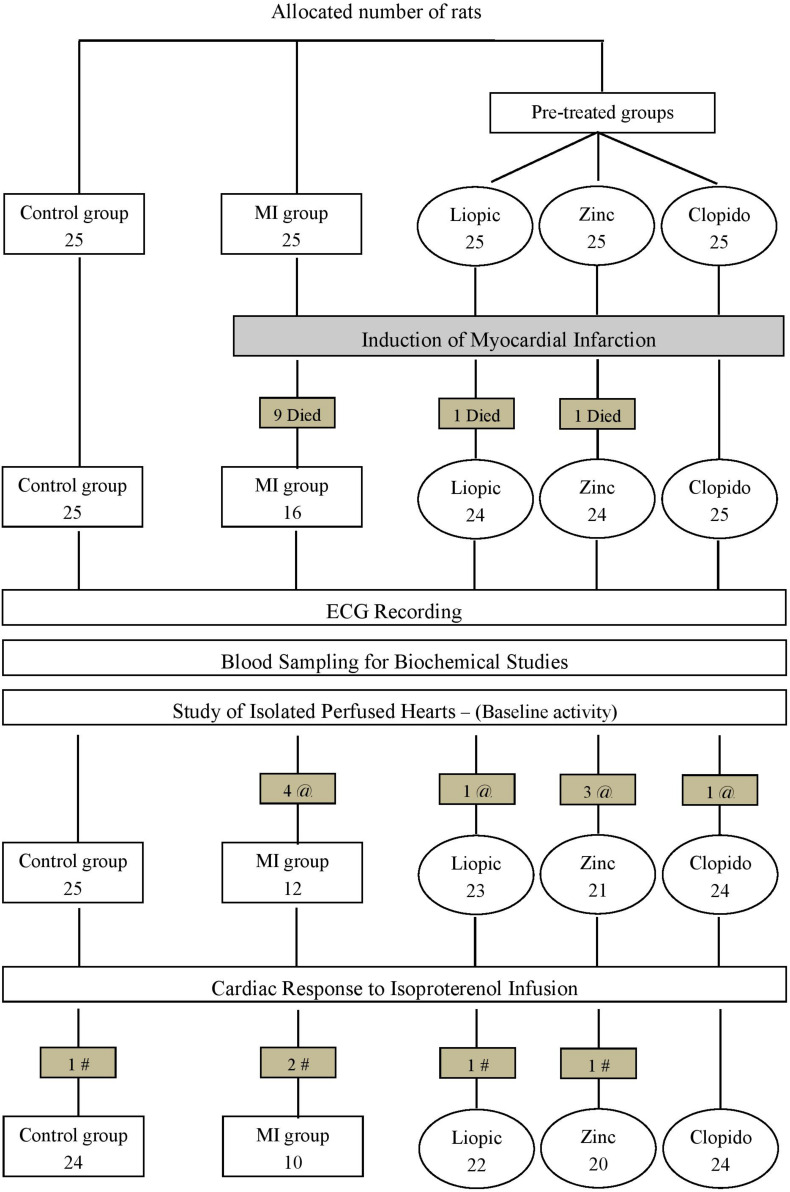
Representative scheme of the experimental protocol. MI, myocardial infarction; Clopido, clopidogrel; @, number of rats with basal arrhythmia; #, number of rats with isoproterenol-provoked arrhythmia.

The present study was performed on Wistar rats of both sexes (initially weighing 150–170 g) that were purchased from the Research Institute of Ophthalmology (Giza, Egypt). Rats were maintained at the Physiology Department Animal House, under controlled conditions (temperature, 23 ± 2°C; humidity, 55 ± 10%; and lighting, 07:00 to 19:00 h) with food and water *ad libitum*. Rats were equally allocated into the following 5 groups (Figure 1):

(1) Control rats: injected subcutaneously with distilled water (the solvent of isoproterenol) for 2 successive days.
(2) Untreated MI rats: subjected to induction of MI by subcutaneous injection of 85 mg/kg isoproterenol (ISO) for 2 successive days at a 24-h interval. ISO was supplied as a powder (Isoprenaline hydrochloride; Sigma-Aldrich, St. Louis, MO, United States) and dissolved in distilled water (El-Salamony and Salman, 2009).
(3) Lipoic acid pre-treated MI rats: received alpha lipoic acid in a dose of 50 mg/kg/day by gavage for 10 days (Thaakur and Himabindhu, 2009). Lipoic acid was supplied as a powder (EVA Pharma for Pharmaceuticals and Medical Appliances SAE, Egypt) and suspended in 0.2% carboxymethyl cellulose (CMC) solution in a concentration of 5 mg/ml. Rats were then subjected to induction of MI on the 9th and 10th days by injection of ISO.
(4) Zinc pre-treated MI rats: received intraperitoneal injections of zinc sulfate in a dose of 80 mg/kg/day for 10 days (Troskot et al., 1997). Zinc sulfate was supplied as a powder (El-Nasr Company, Egypt) and dissolved in normal saline in a concentration of 80 mg/ml. Rats were then subjected to induction of MI on the 9th and 10th days by injection of ISO.
(5) Clopidogrel pre-treated MI rats: received clopidogrel in a dose of 30 mg/kg/day by gavage for 10 days (Uemura et al., 2006). Tablets containing clopidogrel (75 mg, Thrombo; kindly gifted by Egyptian International Pharmaceutical Industries Company, EIPICO) were ground into fine powder and suspended in 0.5% CMC solution in a concentration of 3 mg/ml. Rats were then subjected to induction of MI on the 9th and 10th days by injection of ISO.

### Experimental Procedures

On the day of the experiment (i.e., 24-h after injection of the second dose of ISO), overnight fasted rats were weighed and injected intraperitoneally with 5,000 IU/kg of heparin sodium (Nile Pharmaceutical Co., Cairo, Egypt) (Ahmed, 2013). Half-an-hour later, the rats were anesthetized with intraperitoneal injection of pentobarbital (40 mg/kg). ECG was recorded using cardimax FX-121 (Fukuda Denshi Co. Ltd., Japan). A midline abdominal incision was made, and the abdominal aorta was exposed and cannulated for blood samples collection. Then, the thoracic cavity was opened; the heart was exposed, excised quickly and placed immediately in ice-cold Krebs–Henseleit-Bicarbonate (KHB) buffer solution for fast cardioplegia.

#### Perfusion of Isolated Hearts

Isolated hearts were perfused according to the technique of Langendorff that was described by Ayobe and Tarazi (1983). The heart that stopped beating was rapidly mounted on to a Langendorff apparatus and perfused with KHB buffer (pH 7.4) bubbled with 95% O_2_ – 5% CO_2_, and warmed to 37°C with constant perfusion pressure of 55 mmHg without recirculation. The tension developed by the heart was measured by light weight (1–30 g range) isometric force transducer (K-30 Hugo Jachs Elektronik, Germany), which is connected through a strain gauge (half-bridged Bioscience FC–117 coupler) to a two-channel oscillograph (Washington MD2 Bioscience, United States). The heart was left to stabilize for 15 min. under resting tension of 1 g and then a baseline recording was obtained.

Isoproterenol (ISO) infusion was used to examine the cardiac responsiveness to progressive adrenergic stimulation and to assess the intrinsic cardiac reserve mechanisms. Isoproterenol (Isoprenalina; Egy-Drug, Cairo, Egypt) was provided as an ampoule (0.2 mg/ml) and diluted in KHB buffer to reach a final concentration of 2 μg/ml. It was infused through a catheter tube (PE-50; Clay Adams, New Jersey, United States), connected to an opening just above the aortic cannula using a Sage-355 infusion pump at sequential rates of 0.15, 0.2, and 0.3 ml/min. Each dose level was infused for 3 min. and the recording of cardiac activity was obtained at the end of the third minute.

Baseline (pre-infusion) cardiac activity, responses to each dose of ISO and maximal responses (the highest response to three doses) were recorded and the delta changes (i.e., difference between maximal response and baseline pre-infusion value) were also calculated. In each record, heart rate (HR), peak developed tension (PT), time to peak tension (TPT) and half relaxation time (1/2RT) were determined. Simultaneously the myocardial flow rate (MFR) was assessed by collecting the fluid for 3 min. The PT and MFR both were calculated per 100 mg left ventricular (LV) weight (PT/LV and MFR/LV, respectively).

After perfusion, the hearts were washed with normal saline, blotted by filter paper and weighed in five-digit precision balance (Mettler, AE163). Weights of left ventricle (LV) and whole heart (WH) were expressed as absolute (mg) as well as relative weights normalized to body weight (mg/g).

#### Biochemical Studies

Blood samples were centrifuged at 4,000 rpm for 15 min. and the separated plasma was stored frozen at −20°C for subsequent determination of troponin I, creatine kinase-MB (CK-MB) unit, triglyceride (TG) and total cholesterol (TC) levels. Plasma troponin I levels were estimated by the microplate immunoenzymometric assay (Apple et al., 1999), using kits supplied by Monobind Inc. (Lake Forest, CA, United States). Levels of CK-MB unit (Wu and Bowers, 1982), TG (Scheletter and Nussel, 1975), and TC (Richmond, 1973) were estimated according to enzymatic colorimetric technique using kits supplied by Stanbio Laboratory (San Antonio, TX, United States).

#### Myocardial Oxidative Stress

Oxidative stress in myocardial tissue was assessed by measuring levels of malondialdehyde to detect lipid peroxidation products, as well as levels of anti-oxidant enzymes (superoxide dismutase and catalase) to evaluate the anti-oxidant capacity. Left ventricular homogenates (10%) were prepared; in 50 mM potassium phosphate (pH 7.5) for malondialdehyd (MDA) estimation; in 100 mM potassium phosphate (pH 7.0) containing 2 mM EDTA for superoxide dismutase (SOD) estimation; and in 50 mM potassium phosphate (pH 7.4) containing 1 mM EDTA and 1 ml/l Triton X-100 for catalase (CAT) estimation by using tissue homogenizer (Ultra–Turrax, IKA–Werke GmbH & Co. KG, Staufen, Germany). The homogenates were then centrifuged at 4,000 rpm for 15 min and the clear supernatants were used for the assay of MDA, SOD, and CAT by enzymatic colorimetric technique described by Nishikimi et al. (1972); Ohkawa et al. (1979), and Aebi (1984) respectively; using kits supplied by Bio-diagnostic, Egypt.

### Statistical Analysis

All statistical data and statistical significances were performed using statistical package for social science (SPSS Inc., Chicago, IL, United States) version 16. Statistical significance for differences between the two groups was determined using Student’s *t*-test for parametric unpaired data and the Mann–Whitney test for non-parametric unpaired data. Pearson’s χ^2^ was used for comparison between different groups as regards the incidence of mortality and arrhythmias. A *p*-value less than 0.05 was considered statistically significant.

## Results

### Incidence of Mortality

**FIGURE 2:**
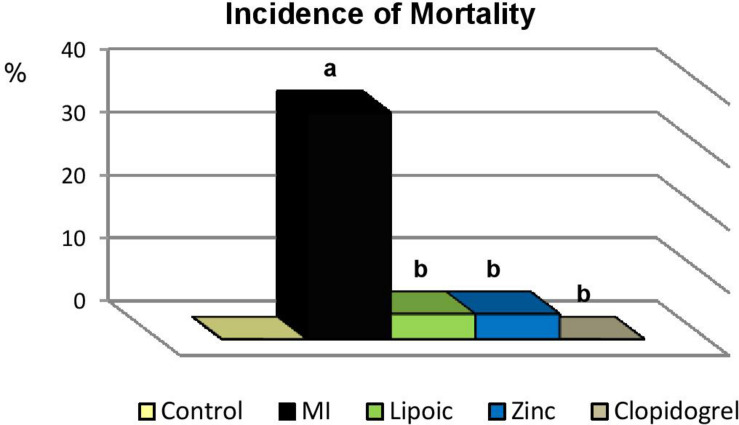
Incidence of mortality in the control, untreated myocardial infarction (MI) and pre-treated (lipoic acid, zinc sulfate and clopidogrel) groups. Significance of differences is calculated by χ^2^-test at *p* < 0.05; a—vs. controls, b—vs. MI group.

As shown in Table 1 and Figure 2, the incidence of MORTALITY was found to be significantly increased in rats with untreated MI compared to their marching controls. Meanwhile, it was significantly reduced with all pre-treatment modalities compared to the untreated MI group.

**TABLE 1:** Incidence of mortality in the control, untreated MI, and pre-treated MI groups.

	Control	Untreated MI	Lipoic acid	Zinc	Clopidogrel
Total number of rats	25	25	25	25	25
Number of dead rats	0	9^a^	1^b^	1^b^	0^b^
Percent ratio of mortality	0%	36%	4%	4%	0%

*MI, myocardial infarction. ^*a*^Significance of differences from control group calculated by χ^2^-test at *p* < 0.05. ^*b*^Significance of differences from MI group calculated by χ^2^-test at *p* < 0.05.*

### ECG Changes

**FIGURE 3:**
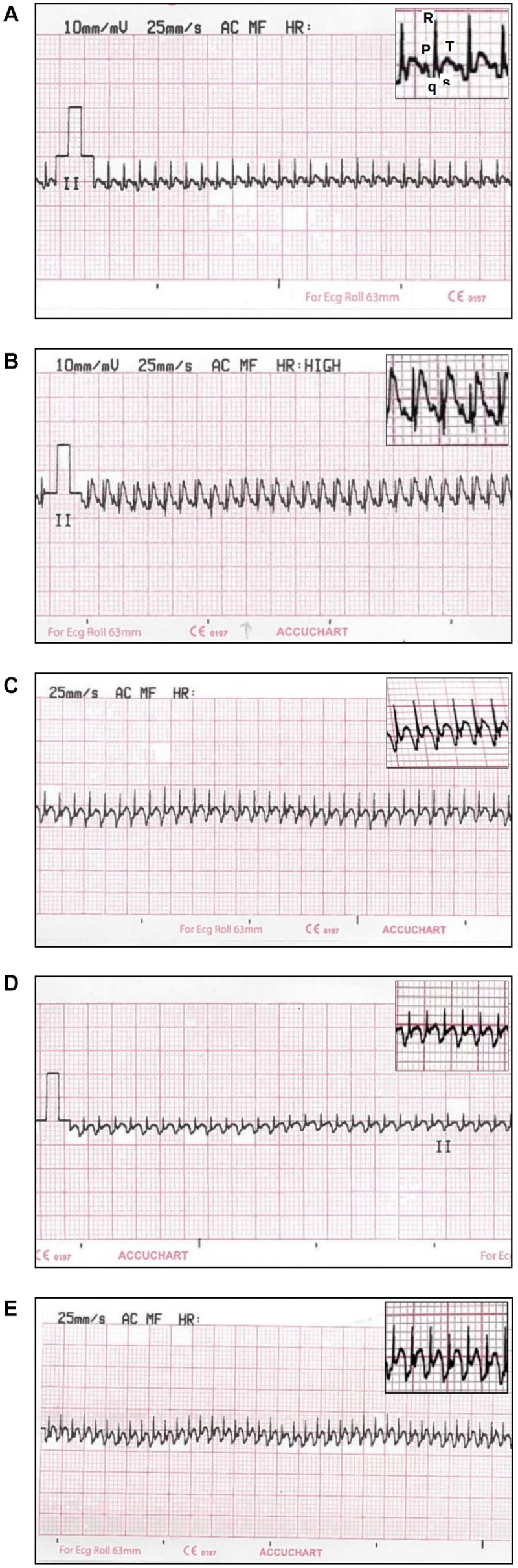
Electrocardiogram (ECG) traces (Lead II) recorded from: **(A)** controls showing regular ECG pattern with defined P, QRS, and T waves; **(B)** ISO-induced MI showing ST-segment elevation; **(C)** lipoic pre-treated rats showing a marked decrease in ST-segment elevation; **(D)** zinc pre-treated rats showing a marked decrease in ST-segment elevation and R wave amplitude; **(E)** clopidogrel pre-treated rats showing a decrease in ST-segment elevation.

The MI-induced elevation in ST-segment was significantly decreased with the three different pre-treatment models to reach the values observed in control group, except for the clopidogrel pre-treated rats which were still significantly higher than controls. HR was only significantly increased with clopidogrel pre-treatment compared to control rats (Table 2 and Figure 3).

**TABLE 2:** Electrocardiographic parameters of the control, untreated MI, and pre-treated MI groups.

	Control	Untreated MI	Lipoic acid	Zinc	Clopidogrel
	(25)	(16)	(24)	(24)	(25)
ST-segment (mm)	0.63 ± 0.11	1.75 ± 0.14^a^	0.90 ± 0.13^b^	0.90 ± 0.18^b^	1.07 ± 0.07^ab^
HR (bpm)	406.75 ± 18.79	419.61 ± 16.10	426.00 ± 12.53	439.93 ± 17.63	461.60 ± 15.35^a^
QTo (msec)	81.25 ± 2.21	116.19 ± 4.05^a^	125.33 ± 3.63^a^	102.67 ± 3.84^ab^	86.67 ± 2.52^b^
QTc (msec)	210.81 ± 8.50	275.33 ± 17.02^a^	335.33 ± 9.79^ab^	277.93 ± 13.25^a^	238.33 ± 6.63^ab^
QRS duration (msec)	23.75 ± 2.02	28.10 ± 2.03	37.33 ± 1.82^ab^	48.00 ± 1.60^ab^	33.33 ± 2.52^a^
QRS voltage (μvolt)	478.13 ± 31.61	473.81 ± 26.42	471.33 ± 53.21	255 ± 38.16^ab^	501.67 ± 32.04

**Values are represented as mean ± SEM. The number of observations is given in parentheses. MI, myocardial infarction; HR, heart rate; QTo, QT interval observed; QTc, QT interval corrected. ^*a*^Significance of differences from control group calculated by Student’s t-test for unpaired data at p < 0.05. ^*b*^Significance of differences from MI group calculated by Student’s t-test for unpaired data at p < 0.05*.*

The significantly prolonged QT interval observed in untreated MI group, compared to controls, was still prominent in lipoic acid pre-treated rats. While both zinc and clopidogrel pre-treatment resulted in significant shortening of QT interval compared to untreated MI group that reached the control values only in clopidogrel pre-treated rats. Upon correction of the QT (QTc) interval for the effect of HR, there was a significant prolongation in untreated MI group as well as all pre-treatment modalities compared to control rats. Lipoic acid pre-treatment resulted in significant prolongation of QTc interval even more than untreated MI rats (Table 2).

The QRS duration showed significant prolongation in all pre-treated groups compared to control rats. It was even, also, significantly prolonged in lipoic acid and zinc pre-treated rats compared to untreated MI group. While the QRS voltage was only significantly reduced in zinc pre-treated rats compared to both control and untreated MI groups (Table 2).

### Cardiac Enzymes and Lipid Profile

Plasma troponin I was significantly elevated in untreated MI rats (33.33%) compared to their matching controls. Upon pre-treatment with lipoic acid and zinc, troponin I level was still significantly higher than controls. Whereas, clopidogrel pre-treatment revealed insignificant results compared to both control as well as untreated MI rats. Levels of CK-MB unit were significantly elevated in untreated MI group (62.16%) compared to controls. Pre-treatment with lipoic acid or clopidogrel produced significant reduction in CK-MB unit to reach the values of control rats. Meanwhile, MI rats pre-treated with zinc still have significantly higher CK-MB unit compared to controls (Table 3).

**TABLE 3:** Plasma levels of troponin I, creatine kinase-MB, triglycerides and total cholesterol in the control, untreated MI, and pre-treated MI groups.

	Control	Untreated MI	Lipoic acid	Zinc	Clopidogrel
	(25)	(16)	(24)	(24)	(25)
Troponin I (ng/ml)					
Median	0.6250	0.8333^a^	0.8542^a^	0.8542^a^	0.7292
25th percentile	0.3333	0.5000	0.4479	0.5209	0.5313
75th percentile	0.7500	1.4375	1.5935	1.07275	1.31275
CK-MB (mU/ml)	280.12 ± 35.60	454.24 ± 61.62^a^	255.33 ± 33.49^b^	614.13 ± 77.56^a^	216.53 ± 32.46^b^
TG (mg/dl)	123.25 ± 8.87	149.67 ± 13.61	77.07 ± 5.38^ab^	150.73 ± 15.98	94.67 ± 6.99^ab^
TC (mg/dl)	78.52 ± 14.21	101.53 ± 7.35	73.40 ± 5.75^b^	81.00 ± 4.80^b^	92.73 ± 4.70

**Values are represented as mean ± SEM (except for troponin I). The number of observations is given in parentheses. MI, myocardial infarction; CK-MB, creatine kinase-MB; TG, triglycerides; TC, total cholesterol. ^*a*^Significance of differences from control group calculated by the Mann–Whitney test in case of troponin I and Student’s t-test for unpaired data in case of CK-MB, TG and TC at p < 0.05. ^*b*^Significance of differences from MI group calculated by Student’s t-test for unpaired data at p < 0.05*.*

Upon comparing the levels of TG and TC, there was no statistical difference among untreated MI and their matching controls. However, plasma TG was significantly lower in MI rats pre-treated with lipoic acid or clopidogrel compared to both control and untreated MI rats. Levels of TC were also significantly lower in lipoic acid and zinc pre-treated rats than untreated MI rats (Table 3). Zinc pre-treatment reduced the high levels of TC in untreated MI rats, but failed to modify the hypertriglyceridemia. This hypocholesterolemic effect could be related to the stabilizing effect of zinc on cardiac membranes where cholesterol is being removed from the blood by the heart tissue and incorporated in the cardiac membranes (Wald and Law, 1995). The reduction in plasma lipid risk factors upon clopidogrel pre-treatment could be ascribed to the hypolipidemic action of anti-oxidants (Yogeeta et al., 2006; Abhilash et al., 2011), as Kanko et al. (2005) presented evidence for the beneficial effect of clopidogrel on re-perfusion injury via inhibition of free radicals.

### Incidence of Arrhythmia in Isolated Hearts

**FIGURE 4:**
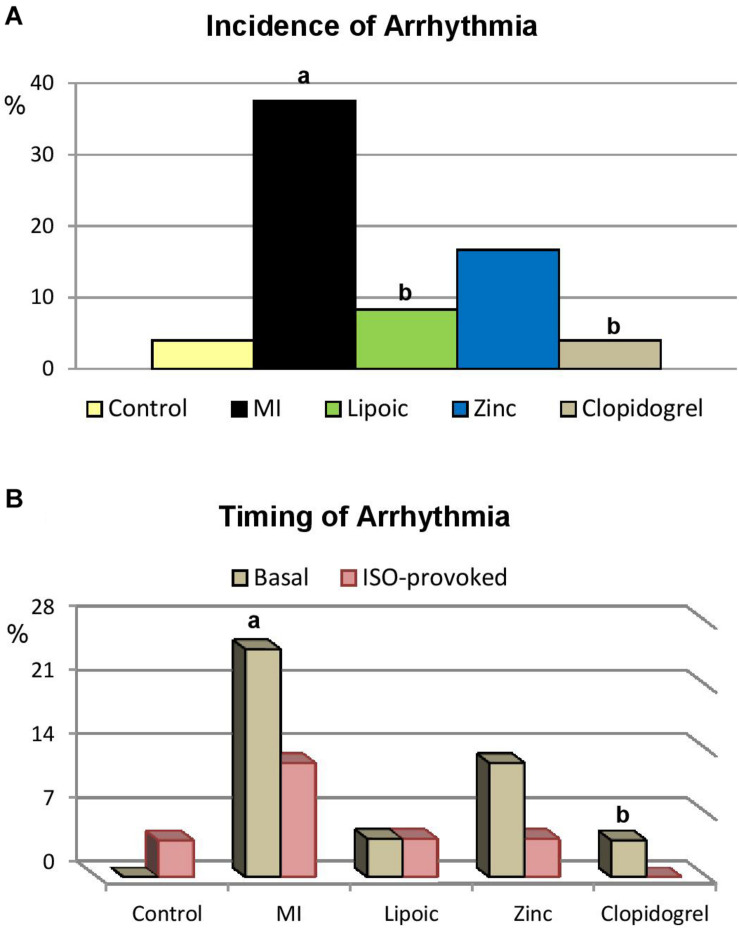
Arrhythmia in the control, untreated myocardial infarction (MI) and pre-treated (lipoic acid, zinc sulfate and clopidogrel) groups. Significance of differences is calculated by χ^2^-test at *p* < 0.05; a—vs. controls, b—vs. MI group; **(A)** incidence of total arrhythmia, **(B)** incidence of each type of the observed arrhythmia; whether at basal condition (Basal) or induced by ISO infusion (ISO-provoked).

Table 4 and Figure 4 show that the incidence of total ARRHYTHMIA was significantly increased in hearts of untreated MI compared to hearts of their matching controls. In the meantime, it was statistically indifferent in lipoic acid and clopidogrel pre-treated hearts compared to controls and much lower than that of untreated MI group, where it reached the level of significance. Similarly, it was unchanged in hearts pre-treated with zinc compared to controls and almost indifferent from hearts of untreated MI group.

**TABLE 4:** Incidence of arrhythmia in perfused hearts of the control, untreated MI, and pre-treated MI groups.

	Control	Untreated MI	Lipoic acid	Zinc	Clopidogrel
Total number of rats	25	16	24	24	25
Number of rats with basal arrhythmia	0	4^a^	1	3	1^b^
Percent ratio	0%	25%	4.17%	12.5%	4%
Number of rats with ISO-provoked arrhythmia	1	2	1	1	0
Percent ratio	4%	12.5%	4.17%	4.17%	0%
Total number of rats with arrhythmia	1	6^a^	2^b^	4	1^*b*^
Percent ratio	4%	37.5%	8.33%	16.67%	4%

**MI, myocardial infarction; ISO, isoproterenol. ^*a*^Significance of differences from control group calculated by χ^2^-test at p < 0.05. ^*b*^Significance of differences from MI group calculated by χ^2^-test at p < 0.05*.*

### Intrinsic Cardiac Activities of Isolated Perfused Hearts

Although, untreated MI group had significant baseline bradycardia, they demonstrated significantly higher delta change in response to isoprotrenol (ISO) infusion compared to their matching controls. Baseline TPT was significantly prolonged with higher delta change in response to graded ISO infusion compared to controls. Contractile function (PT/LV), 1/2 RT and MFR/LV; baseline value, maximal response and delta change were all insignificant from control rats (Table 5).

**TABLE 5:** Intrinsic cardiac activities of perfused isolated hearts from the control, untreated MI, and pre-treated MI groups.

	Control	Untreated MI	Lipoic acid	Zinc	Clopidogrel
**HR (bpm)**					
Baseline values	220.33 ± 13.10(25)	157.95 ± 13.90^a^(12)	147.86 ± 16.30^a^(23)	172.67 ± 16.39^a^(21)	177.64 ± 19.29(24)
Maximal responses	216.76 ± 11.72(24)	199.82 ± 12.86*(10)	150.38 ± 18.56^ab^(22)	201.09 ± 18.60*(20)	227.43 ± 12.53*(24)
Delta changes	−1.06 ± 7.75(24)	51.47 ± 9.61^a^(10)	8.62 ± 13.20^b^(22)	39.45 ± 11.85^a^(20)	49.79 ± 17.24^a^(24)
**PT/LV (g/100 mg)**					
Baseline values	0.99 ± 0.16(25)	0.73 ± 0.10(12)	0.81 ± 0.19(23)	0.73 ± 0.10(21)	1.11 ± 0.11^ab^(24)
Maximal responses	1.06 ± 0.10*(24)	0.87 ± 0.12*(10)	0.68 ± 0.09^ab^(22)	0.74 ± 0.09^a^(20)	1.18 ± 0.10^ab^(24)
Delta changes	0.21 ± 0.08(24)	0.17 ± 0.03(10)	0.04 ± 0.02^ab^(22)	0.11 ± 0.06(20)	0.07 ± 0.04^a^(24)
**TPT (msec)**					
Baseline values	63.89 ± 5.89(25)	92.63 ± 6.30^a^(12)	73.57 ± 6.76(23)	59.17 ± 5.14^b^(21)	122.14 ± 11.63^ab^(24)
Maximal responses	52.94 ± 4.18*(24)	67.06 ± 5.74*(10)	70.77 ± 6.25^a^(22)	46.36 ± 3.10*^b^(20)	85.71 ± 8.37*^a^(24)
Delta changes	−10.00 ± 4.46(24)	−27.06 ± 5.99^a^(10)	−5.38 ± 5.38^b^(22)	−14.55 ± 5.29(20)	−36.43 ± 10.30^a^(24)
**1/2 RT (msec)**					
Baseline values	45.56 ± 3.90(25)	38.95 ± 2.41(12)	40.00 ± 2.57(23)	38.33 ± 3.45(21)	42.86 ± 2.66(24)
Maximal responses	32.35 ± 2.19*(24)	26.47 ± 2.09*(10)	34.62 ± 3.51*^b^(22)	28.18 ± 2.26*(20)	32.86 ± 2.44*(24)
Delta changes	−14.12 ± 2.98(24)	−13.53 ± 2.56(10)	−5.38 ± 2.43^ab^(22)	−10.00 ± 2.70(20)	−10.00 ± 2.57(24)
**MFR/LV (ml/min/100 mg)**					
Baseline values	2.23 ± 0.26(25)	2.07 ± 0.24(12)	1.96 ± 0.17(23)	3.58 ± 0.57^ab^(21)	2.18 ± 0.20(24)
Maximal responses	2.30 ± 0.28(24)	2.13 ± 0.27(10)	1.81 ± 0.17(22)	3.60 ± 0.68^b^(20)	2.56 ± 0.28*(24)
Delta changes	0.16 ± 0.09(24)	0.16 ± 0.11(10)	−0.05 ± 0.07(22)	0.02 ± 0.14(20)	0.37 ± 0.12(24)

Values are represented as mean ± SEM. The number of observations is given in parentheses. MI, myocardial infarction; HR, heart rate; PT/LV, peak tension/left ventricle; TPT, time to peak tension; 1/2RT, half-relaxation time; MFR/LV, myocardial flow rate/left ventricle. ^*a*^Significance of differences from control group calculated by Student’s t-test for unpaired data at p < 0.05. ^*b*^Significance of differences from MI group calculated by Student’s t-test for unpaired data at p < 0.05. * Significance of the maximal response to baseline value of the same group calculated by Student’s t-test for paired data at p < 0.05.

#### LIPOIC Acid Pre-treated MI

LIPOIC acid pre-treated MI rats showed significant baseline bradycardia with lowered maximum response to graded ISO infusion compared to controls. At the same time, this maximal response and the delta change were also significantly lowered when compared to untreated MI rats. The baseline PT/LV was statistically in different from controls and untreated MI, though the maximal response and delta change were significantly reduced. Baseline TPT was statistically indifferent compared to both control and untreated MI rats. However, the maximal TPT was significantly prolonged compared to controls only with significantly less delta change compared to untreated MI rats. Although baseline 1/2 RT was indifferent, the maximal value was significantly prolonged with significantly reduced delta change compared to both control and untreated MI rats. No statistical difference could be detected in MFR/LV baseline value, maximal response or even delta change on comparing lipoic acid pre-treated rats with either control or untreated groups (Table 5). The absence of significant differences between rats pre-treated with LA and those untreated regarding; basal HR, PT/LV, TPT, 1/2 RT, and MFR/LV, indicate that LA pre-treatment did not alter the ISO effects on intrinsic functions of isolated hearts. Yet, upon β-adrenergic stimulation, MI hearts pre-treated with LA showed more deterioration of the maximal inotropic responsiveness and inotropic reserves compared to hearts from untreated MI rats. Such deterioration in inotropic abilities could be attributed to absence of left ventricular hypertrophy (LVH) which represents an important compensatory mechanism.

#### ZINC Pre-treated MI

ZINC pre-treated MI rats displayed significant baseline bradycardia with higher delta change, in response to graded ISO infusion, compared only to control rats. Regarding PT/LV, only the maximal response was significantly reduced compared to control rats. Meanwhile, the baseline TPT and maximal response were significantly shorter than untreated MI rats, the 1/2 RT didn’t show any statistical differences. Notably, baseline MFR/LV was significantly higher with zinc pre-treatment compared to both control and untreated MI groups. Though the maximal value of MFR/LV was still higher, it reached the level of significance only when compared to untreated MI rats (Table 5). Zinc pre-treated MI rats did not show any differences from those of untreated MI rats as regard their basal chronotropy as well as their maximal responsiveness to β-adrenergic stimulation, and their chronotropic reserve was preserved. The basal inotropy, measured as PT/LV, was similar to that of hearts with untreated MI, but their maximal responsiveness to β-adrenergic stimulation and inotropic reserves were reduced, though they didn’t reach level of significance. These data, therefore, indicate that zinc pre-treatment of ISO-induced MI results in deterioration in inotropic abilities and reserves, which represents a serious state of systolic dysfunction. In accordance, Schwarz et al. (2009) reported that in chronic coronary occlusion of rat heart, zinc did not improve the ejection fraction compared to controls, and could not reduce the apoptotic cell death in myocardial infarction.

#### CLOPIDOGREL Pre-treated MI

Heart rate of the CLOPIDOGREL pre-treated MI rats revealed no statistical differences when compared to both controls and untreated MI rats, except for the significantly higher delta change compared to control rats. Baseline inotropic activity, PT/LV, and the maximal contractile response to graded ISO infusion were significantly increased with clopidogrel pre-treatment compared to both control and untreated MI groups, with less delta change compared to control rats only. The baseline TPT was significantly prolonged compared to both control and untreated MI groups, while the maximal value was significantly prolonged associated with high delta change compared to controls only. No statistical difference could be detected in 1/2 RT and MFR/LV; baseline value, maximal response or even delta change on comparison with either control or untreated MI groups (Table 5). Isolated perfused hearts from clopidogrel pre-treated MI rats maintained their intrinsic chronotropy and maximal chronotropic responsiveness and preserved their chronotropic reserves. Concerning intrinsic inotropic activity, these hearts revealed augmented ability to generate force, as peak developed tension, whether expressed as total PT or per each LV contractile unit. Also, the maximal inotropic responses to β-adrenergic stimulation were significantly increased. Such maintained inotropic ability, both basal and during stress, actually represents the most important effective functional compensatory mechanism which could be underlying the absence of mortality and 100% survival in this group of rats. This can be supported by Liu et al. (2003) who considered that left ventricular dysfunction and ventricular arrhythmia are the two determinants for predicting the mortality after acute MI.

### Left Ventricular Weight Changes

**FIGURE 5:**
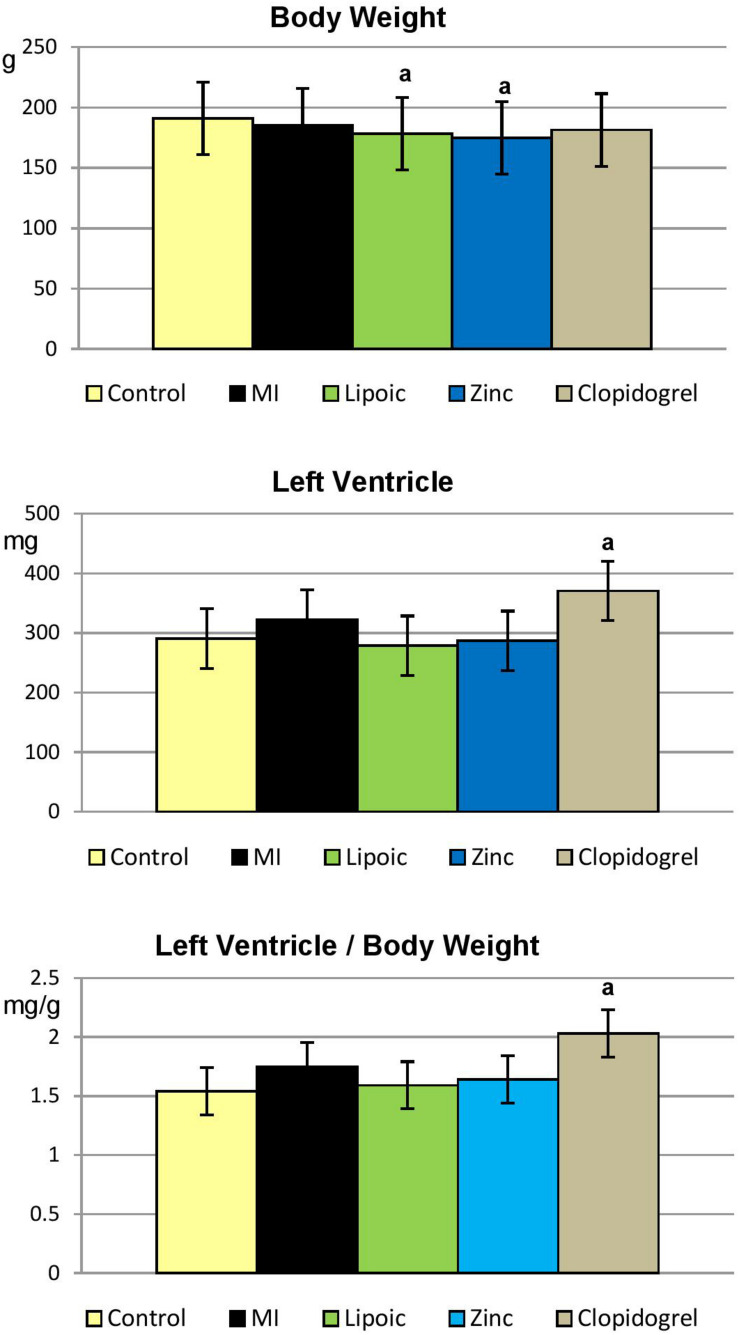
Absolute left ventricular weight and its ratio to body weight in the control, untreated myocardial infarction (MI) and pre-treated (lipoic acid, zinc sulfate, and clopidogrel) groups. Results are represented as mean ± SEM, significance of differences is calculated by Student’s *t*-test for unpaired data at *p* < 0.05; a—vs. controls.

As shown in Table 6 and Figure 5, lipoic acid and zinc pre-treated MI rats showed significant reduction in their body weight compared to control rats. Remarkably, clopidogrel pre-treated MI group demonstrated significant increase in LV and LV/BW ratio compared only to control rats.

**TABLE 6:** Body weight, left ventricular weight and left ventricular body weight ratio in the control, untreated MI, and pre-treated MI groups.

	Control	Untreated MI	Lipoic acid	Zinc	Clopidogrel
	(25)	(16)	(24)	(24)	(25)
BW (g)	190.95 ± 3.78	185.52 ± 4.07	178.20 ± 4.65^a^	174.80 ± 4.33^a^	181.33 ± 4.11
LV (mg)	290.60 ± 19.85	322.00 ± 24.65	278.53 ± 14.07	286.66 ± 31.19	370.76 ± 29.11^a^
LV/BW (mg/g)	1.54 ± 0.11	1.75 ± 0.14	1.59 ± 0.11	1.64 ± 0.17	2.03 ± 0.14^a^

**Values are represented as mean ± SEM. The number of observations is given in parentheses. MI, myocardial infarction; BW, body weight; LV, left ventricular weight; LV/BW, left ventricular body weight ratio. ^*a*^Significance of differences from control group calculated by Student’s t-test for unpaired data at p < 0.05*.*

### Myocardial Tissue Oxidative Stress Parameters

**FIGURE 6:**
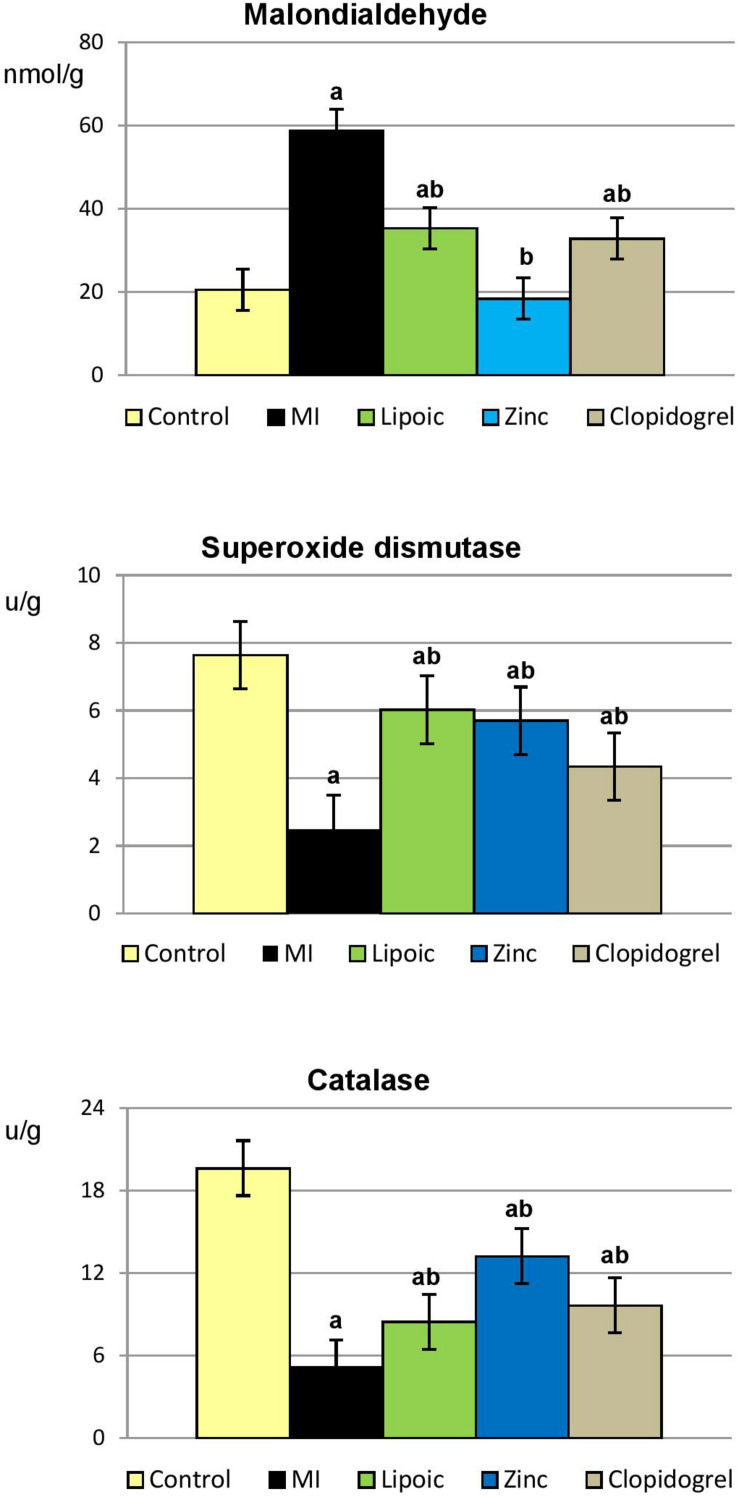
Myocardial tissue oxidative stress parameters in the control, untreated myocardial infarction (MI) and pre-treated (lipoic acid, zinc sulfate, and clopidogrel) groups. Results are represented as mean ± SEM, significance of differences is calculated by Student’s *t*-test for unpaired data at *p* < 0.05; a—vs. controls, b—vs. MI group.

Induction of MI resulted in significantly elevated levels of MDA, end product of lipid peroxidation and marker for oxidative stress, accompanied with significant decrease in the activities of anti-oxidative enzymes (SOD and CAT) in myocardial tissue as compared to control rats. However, pre-treatment with lipoic acid, zinc and clopidogrel significantly decreased the levels of MDA and increased the activities of these anti-oxidants as compared to their respective ISO-induced MI rats. Interestingly, the reduction of MDA level in myocardial tissue of zinc pre-treated rats reached the level of normal controls (Table 7 and Figure 6).

**TABLE 7:** Cardiac tissue oxidative stress parameters of the control, untreated MI, and pre-treated MI groups.

	Control	Untreated MI	Lipoic acid	Zinc	Clopidogrel
	(25)	(16)	(24)	(24)	(25)
MDA (nmol/g)	20.56 ± 1.29	58.84 ± 3.40^a^	35.34 ± 1.63^*ab*^	18.42 ± 1.36^b^	32.85 ± 1.94^*ab*^
SOD (μ/g)	7.64 ± 0.49	2.50 ± 0.30^a^	6.03 ± 0.33^*ab*^	5.70 ± 0.30^*ab*^	4.35 ± 0.18^*ab*^
CAT (μ/g)	19.61 ± 1.39	5.11 ± 0.34^a^	8.45 ± 0.42^*ab*^	13.20 ± 1.21^*ab*^	9.63 ± 0.64^*ab*^

**Values are represented as mean ± SEM. The number of observations is given in parentheses. MI, myocardial infarction; MDA: malondialdehyde; SOD, superoxide dismutase; CAT, catalase.^*a*^Significance of differences from control group calculated by Student’s t-test for unpaired data at p < 0.05. ^*b*^Significance of differences from MI group calculated by Student’s t-test for unpaired data at p < 0.05*.*

## Discussion

Isoproterenol (ISO)-induced myocardial infarction (MI) has been widely used as a successful experimental non-invasive model of MI, that results in myocardial alterations similar to those observed in MI in human beings (Farvin et al., 2004; Díaz-Muñoz et al., 2006). The results obtained in the present study agree with the main criteria generally used for the documentation of MI, mainly ST-segment elevation in ECG, and the rise of serum levels of cardiac marker enzymes and troponins. The study also revealed the outcomes of receiving cardioprotective agents concomitant with the induction of MI.

### LIPOIC Acid Pre-treatment

LIPOIC acid (LA) pre-treatment exerted a protective effect against ISO-induced MI, as evidenced by significant reduction in ST-segment elevation. Such effect could be ascribed to the solubility of LA in membranous and aqueous phases, thus effectively preventing the damage of cell membranes by lipid peroxides (Mythili et al., 2004). Meanwhile, protection was also evident by normalization of plasma level of CK-MB, the level of troponin I was still significantly elevated above the control value and indifferent from that of untreated MI, denoting incomplete protection. Such partial protection was sufficient to reduce the percent mortality and incidence of arrhythmias to low levels indifferent from control rats. Our results document the anti-oxidant effect of LA which was in agreement with Makeeva et al. (2008) who reported that LA is effective in protecting the heart and preventing the development of free radical oxidation during myocardial ischemia in rats. Also, Kumazaki et al. (2011) showed that LA protects against the arsenic trioxide-induced acute cardiac toxicity and subsequent cardiac death in rats. On the other hand, Ghibu et al. (2012) documented that administration of LA had no beneficial effects on doxorubicin-induced cardiotoxicity in rats.

Further, LA pre-treatment resulted in a hypolipidemic effect on plasma TG and TC, thus decreasing the contribution of these high risk factors to the pathogenesis of ISO-induced MI. This significant reduction in plasma levels of TG and TC upon LA treatment could be ascribed to the known hypolipidemic action of the anti-oxidative agents (Yogeeta et al., 2006; Abhilash et al., 2011). In contrast, LA-induced decrease in ROS production was responsible for the absence of LVH in these rats, which is claimed for the deteriorated inotropic function observed in this group. Goraca et al. (2009) indicated that LA abrogates cardiac hypertrophy through its action as a free radical scavenger and a potent anti-oxidant, since increased ROS production can result in cardiomyocyte hypertrophy. Another compensatory mechanism that was compromised in LA pre-treated hearts is the relaxing effect of adrenergic stimulation; consequently hearts pre-treated with LA suffered from both systolic and diastolic dysfunctions.

### ZINC Pre-treatment

ZINC pre-treatment seems to have protected the cardiac membranes, as evidenced by normalization of ST-segment, but failed to influence the high levels of circulating markers of injury, namely CK-MB and troponin I. Previous studies have shown that zinc treatment protects against ISO-induced MI due to a stabilizing effect on lysosomal membranes (Chvapil et al., 1972), better membrane integrity related to ATPase and phospholipase A2 inhibition (Chvapil and Owen, 1977), and probably by reducing free radical-induced membrane changes (Singal et al., 1981). The mixed effects of zinc treatment on ECG parameters (normalization of ST-segment, reduced R-voltage and prolonged QRS duration) could be explained by its beneficial effect on the integrity of cardiac membranes, but little or no action on the myocardial damage induced by ISO. In the meantime, although zinc pre-treatment reduced the percent mortality of ISO-treated rats to become statistically indifferent from controls, yet it did not modify the tendency for arrhythmias which remained almost similar to hearts with ISO-induced MI. In contrast, Karagulova et al. (2007) demonstrated that administration of zinc decreased arrhythmias more than two-fold in rodent model of ischemia/re-perfusion. Actually, the protective effect of zinc against MI is controversial. On one hand, zinc sulfate pre-treatment had a potent prophylactic effect limiting the size of MI in rats (Sharma et al., 1987), and in dogs (Lal, 1991), and on the other hand, zinc treatment failed to reduce the infarct size after temporary coronary occlusion followed by re-perfusion and did not demonstrate any reduction in apoptotic cell death (Schwarz et al., 2009).

The compromised inotropic function in zinc pre-treated hearts could be accounted for by the absence of LVH which represents an important compensatory mechanism for force generation. Failure of zinc treatment to reduce apoptotic cell death in hearts with MI (Schwarz et al., 2009) may underlie the absence of LVH. Also, the remarkable anti-oxidative action of zinc, which decreases the production of ROS, could be held responsible for the absence of LVH (Goraca et al., 2009). On the other hand, the rate of contraction measured as TPT was improved upon zinc pre-treatment both under basal conditions and in response to β-adrenergic stimulation and this may compensate for the associated systolic dysfunction as regards force generation. Another compensatory mechanism is the diastolic function; zinc treatment maintained the accelerated rate of relaxation observed in untreated ISO-infracted hearts both under basal conditions and in response to β-adrenergic stimulation. Also, myocardial coronary flow was augmented upon zinc pre-treatment both when calculated as total flow and per each LV contractile unit and this may constitute another compensatory mechanism.

### CLOPIDOGREL Pre-treatment

CLOPIDOGREL pre-treatment revealed that both ST-segment and plasma level of CK-MB were significantly reduced, while plasma troponin I was decreased though insignificantly. These data may, thus, indicate that clopidogrel treatment could control and reduce the ISO-induced myocardial damage, but could not prevent it completely. In accordance, a clinical study demonstrated that clopidogrel treatment reduced the size of MI in patients undergoing primary percutaneous coronary intervention (PPCI) (Song et al., 2012). Meanwhile, the mortality of clopidogrel pre-treated MI rats was actually prevented, being zero percent (0%), indifferent from control rats (0%). Clinical studies are in agreement to our data as indicated by Dörler et al. (2011) who reported that clopidogrel pre-treatment was associated with reduced mortality in PPCI for acute ST-Elevation Myocardial Infarction (STEMI). Arrhythmias were also minimized to 4%, identical to controls (4%). Arrhythmia-reducing effect of clopidogrel in ISO-treated rats could be related to its significant decreasing effect on QT interval. This significant reduction in the incidence of arrhythmias upon clopidogrel treatment could be responsible for its preventing effect on mortality (Liu et al., 2003).

The LVH observed in hearts from clopidogrel pre-treated rats constitutes another compensatory mechanism which adds to the increased inotropic ability. Although the rate of contraction was reduced upon clopidogrel pre-treatment, the maintained relaxation rate and myocardial coronary flow, which were even augmented in response to β-adrenergic stimulation, represent another compensatory mechanism that adds to the enhanced ventricular systolic function. The documented improvement of endothelial function and nitric oxide bioavailability upon clopidogrel treatment (Schäfer et al., 2011) may underlie the augmented coronary flow in this group of rats.

## Conclusion

The different treatment modalities used (lipoic acid, zinc, and clopidogrel) were variably effective in partially modifying the ISO-induced MI insults. All treatments offered partial protection against ISO-induced myocardial damage, evidenced by significant decrease in ST-segment elevation and decrease in plasma level of CK-MB unit with lipoic acid and clopidogrel. In addition, all treatment modalities reduced mortality and were anti-arrhythmogenic except zinc. Also, a hypolipidemic effect which comprised decline in plasma level of TG or TC or both, was exerted by all agents. Clopidogrel favorably produced conservation of cardiac performance and prevention of ischemia, effects that could not be achieved by lipoic acid and zinc treatments.

Despite the beneficial effects of the agents used, none of them was successful in offering complete reversal of all ISO-induced disturbances, nor could any of them alone provide complete protection against the dysfunction inflicted by MI. Therefore, further studies are recommended using a combination of two or more of these agents, which could help to pave the way to achieve useful and effective means for protection against MI.

## Data Availability Statement

The original contributions generated for this study are included in the article/supplementary material, further inquiries can be directed to the corresponding author.

## Ethics Statement

The animal study was reviewed and approved by Research Ethics Committee (REC), Faculty of Medicine, Ain Shams University.

## Author Contributions

EA-H, FM, MoA, MA-S, and MaA contributed to the study conception and design. Material preparation, data collection, and analysis were performed by EA-H, MoA, and MA-S. The first draft of the manuscript was written by EA-H and all authors commented on previous versions of the manuscript. All authors read and approved the final manuscript.

## Conflict of Interest

The authors declare that the research was conducted in the absence of any commercial or financial relationships that could be construed as a potential conflict of interest.
